# Loss or Dilution—A New Diagnostic Method to Assess the Impact of Dilution on Standard Laboratory Parameters

**DOI:** 10.3390/diagnostics13152596

**Published:** 2023-08-04

**Authors:** Nicole Innerhofer, Sasa Rajsic, Marco Ronzani, Robert Breitkopf, Can Gollmann Tepeköylü, Corinna Velik-Salchner, Lisa Schlosser, Dietmar Fries, Werner Streif, Michael Schirmer, Judith Martini

**Affiliations:** 1 Department of Anesthesiology and Intensive Care Medicine, Medical University Innsbruck, 6020 Innsbruck, Austria; nicole.innerhofer@i-med.ac.at (N.I.); dietmar.fries@tirol-kliniken.at (D.F.);; 2 Department for Cardiac Surgery, Medical University of Innsbruck, 6020 Innsbruck, Austria; 3 Department of Mathematics, University of Innsbruck, 6020 Innsbruck, Austria; 4 Department of Paediatrics, Medical University of Innsbruck, 6020 Innsbruck, Austria; 5 Department of Internal Medicine, Medical University of Innsbruck, 6020 Innsbruck, Austria

**Keywords:** dilution, cardiac surgery, biomarkers, hemoglobin, platelets, blood product substitution, volume therapy, fluid therapy

## Abstract

Intraoperative fluid therapy is regularly used in patients undergoing cardiac surgery procedures with cardiopulmonary bypass (CPB). Although fluid administration has several advantages, it unavoidably leads to hemodilution. The hemodilution may further influence the interpretation of concentration-based laboratory parameters like hemoglobin (Hgb), platelet count (PLT) or prothrombin time (PT). These all parameters are commonly used to guide blood product substitution. To assess the impact of dilution on these values, we performed a prospective observational study in 174 patients undergoing elective cardiac surgery. We calculated the total blood volume according to Nadler’s formula, and fluid therapy was correlated with a newly developed dilution coefficient formula at the end of CPB. Intravenously applied fluids were measured from the beginning of the anesthesia (baseline, T_0_) and 15 min after the end of protamine infusion (end of CPB, T_1_). The amount of the administered volume (crystalloids or colloids) was calculated according to the percentage of the intravascular fluid effect, and intraoperative diuresis was further subtracted. The median blood volume increased by 148% in all patients at T_1_ compared to the calculated total blood volume at T_0_. This led to a dilution-dependent decrease of 38% in all three parameters (Hgb 24%, corrCoeff = 0.53; PLT 41%, corrCoeff = 0.68; PT 44%, corrCoeff = 0.54). The dilution-correlated decrease was significant for all parameters (*p* < 0.001), and the effect was independent from the duration of CPB. We conclude that the presented calculation-based approach could provide important information regarding actual laboratory parameters and may help in the guidance of the blood product substitution and potential transfusion thresholds. Further research on the impact of dilution and related decision-making for blood product substitution, including its impact on morbidity and mortality, is warranted.

## 1. Introduction

Perioperative fluid therapy in cardiac surgery is an important factor for maintaining adequate perfusion pressure, blood loss substitution, reducing the demand for catecholamines and preserving microvascular function [1,2]. At present, the guidance for fluid therapy and substitution of blood products during cardiac surgery is mostly based on hemodynamic and laboratory parameters such as mean arterial pressure, hemoglobin (Hgb) or hematocrit (Hct). Whereas different hemodynamic parameters may guide fluid resuscitation, it is not clear to what extent Hgb or Hct values may be adequate to guide blood substitution therapy. This holds especially true in situations of considerable fluid administration with varying degrees of hemodilution, as in the case of cardiac surgery.

Several studies showed that Hct may be used as a surrogate parameter for assessing patient hydration status [2,3,4,5,6]; however, most of these studies could not clearly report the complete volume status of the patient, including all fluid intakes and losses. Therefore, it may be complex to base the decision of whether to substitute blood products or not purely based on the concentration of laboratory variables, such as Hgb or Hct. Moreover, these parameters may be severely affected by the individual patient hydration status. In light of the vast body of the literature showing that the transfusion of packed red blood cells is associated with increased morbidity and mortality after cardiac surgery [7,8,9,10,11], it seems to be of prime importance to define a method of how to help the decision-making and determination of transfusion thresholds, including patient hydration status in the case of hemodilution.

The impact of hemodilution on frequently used laboratory values like Hgb, platelets (PLT) or prothrombin time (PT) have not been examined in a calculated approach so far. The correct assessment of hemodilution in patients undergoing cardiac surgery is complex, and the exact levels of hemodilution may be underestimated. Therefore, the aim of this prospective observational trial is to address the assessment of hemodilution in patients undergoing cardiac surgery.

## 2. Materials and Methods

### 2.1. Study Design

We conducted an observational prospective single-center trial of patients undergoing cardiac surgery between November 2018 and July 2022. We included all patients undergoing elective coronary artery bypass graft surgery and/or valve surgery (limited to a maximum of two valves). Exclusion criteria were salvage or emergency cardiac surgery, patients with endocarditis, single aortic surgery procedures and patients with a known history of coagulopathy, complement deficiency or autoimmune disorders. The immediate postoperative care of patients was utilized at the tertiary intensive care units of the department for anesthesiology and critical care medicine of the Medical University Innsbruck, Austria.

The study was approved by the Ethical committee of the Medical University Innsbruck, Austria (1097/2018) and registered with the ClinTrials.gov (NCT05033236). All patients signed a written informed consent form before the study inclusion.

### 2.2. Blood Sampling and Laboratory Tests

Blood samples were obtained through arterial cannula (BD, Singapore, REF 682245) before the anesthesia induction (baseline, T_0_) and 15 min after the end of protamine infusion (end of CPB, T_1_). The time-point of 15 min after the end of protamine infusion was chosen as it could provide the most accurate information with a rather low probability of significant blood loss thereafter. Thus, the impact of dilution on the measured blood parameters should be more precise as compared to the end of surgery with possible blood loss due to intraoperative bleeding. If no arterial line was available, blood was obtained through a central venous catheter (Arrow International, Reading, PA, USA, REF CS-25854-E), which was inserted for the surgical procedure.

Routine laboratory analyses were performed by the referenced central laboratory unit. All procedures and blood draws were performed by specially trained medical staff for the purposes of this study. Routine institutional care was carried out independent from the blood drawings.

### 2.3. Data Collection, Anesthesia and Cardiopulmonary Bypass Management

We collected the data related to (1) the sociodemographic background of the patient; (2) medical history and ongoing medication; (3) underlying disease and surgery indication; (4) cardiac surgery risk stratification score—Euroscore II [12], (5) perioperative vital parameters; (6) fluid therapy and diuresis (including total colloids and crystalloids and cardiopulmonary bypass positive fluid balance change); (7) blood product substitution (including packed red blood cell units, each of 280 mL and platelet concentrate units, each of 300 mL); (8) surgery duration and (9) concomitant medication. Moreover, we collected the values of Hgb (g/dL), PT (%) and PLT (G/L) at the times T_0_ and T_1_. Data were stored in the local electronic medical record.

General anesthesia was induced with fentanyl (0.2 µg/kg), midazolam (0.1 mg/kg), S-ketamin (25–50 mg) and rocuronium (0.6 mg/kg). For the maintenance of anesthesia, sevoflurane (2.0 Vol. %) and remifentanil were used. Two central venous catheters were placed. For fluid management, balanced crystalloids (Elomel™/Fresenius Kabi Austria, Graz, Austria) or colloids (Gelofusin™/Braun Germany, Melsungen, Germany) were used. Hemodynamic support was provided using the continuous infusion of noradrenalin with a target mean arterial pressure of more than 65 mmHg. When needed, vasopressin was used additionally in some cases (*n* = 14; 8.05%). Inotropic support was provided using milrinone when indicated. The continuous assessment of patient heart function was provided by the anesthesiologist in charge using transesophageal echocardiography. During the surgery, and according to the local operating procedure protocols, all patients received a total of 2 g of tranexamic acid, 400 mg of magnesiumsulfat-heptahydrat and standard antibiotic prophylaxis.

All extracorporeal devices were from the same model unit (Sorin Group S5™; LivaNova GmbH, Munich, Germany), and the same oxygenator (Terumo Capiox™) was used. The priming volume was composed of 800 mL of crystalloid (Elomel™), Mannitol 15% (200–250 mL), 500 mL colloid (Gelofusin™), 1 g of tranexamic acid and 10,000 IU of heparin.

### 2.4. Calculations of Blood Volume and the Individual Dilutional Coefficients

The total blood volume (TBV) was calculated according to the established formula by Nadler et al., the “Nadler-equation”, which had previously been validated using radioisotopic measurements [13]:

(1) $$blood{volume}_{T_{0}}[L]=\{\begin{array}{c} 0.3561\cdot height{\left[m\right]}^{3}+0.03308\cdot weight\left[kg\right]+0.1833,\;\mathrm{for}\;female\;patients\\ 0.3669\cdot height{\left[m\right]}^{3}+0.03219\cdot weight\left[kg\right]+0.6041,\;\mathrm{for}\;male\;patients \end{array}$$

To estimate the impact of intravenous fluids, 25% of the volume of crystalloid fluids and 80% of the volume of colloid fluids were added to the fluid balance. The packed red blood cells contained 280 mL of fluid and 40 g of Hgb, adding up to a mean of 0.9 g/dL hgb, which was in line with the available literature. Every platelet concentrate or fresh frozen plasma (FFP) was balanced with an additional 300 mL or 250 mL per unit, respectively. Finally, the positive fluid balance from the CPB was added. The diuresis was measured at the end of surgery (in mL) and subtracted from the total fluid balance. Lastly, for each patient, the total fluid balance was related to the calculated TBV according to Nadler et al. [13].

Dilution coefficient:

(2) $$DF=\frac{\left(FBC+{Crys}_{T_{0}T_{1}}\cdot 0.25+{Col}_{T_{0}T_{1}}\cdot 0.8+{PRBC}_{T_{0}T_{1}}\cdot 280+{PC}_{T_{0}T_{1}}\cdot 300+{FFP}_{T_{0}T_{1}}\cdot 250-diuresis\right)}{TBV{}_{T_{0}}}$$

*DF*, dilution coefficient; *FBC*, total positive fluid balance from the CPB (in mL); *Crys*, crystalloids; *Col*, colloids; *PRBC*, packed red blood cells; *PC*, platelet concentrate; *FFP*, fresh frozen plasma; *TBV*, total blood volume (mL).

To validate the dilution effect on the laboratory parameters, the older “Allen” equation was used as already proposed by Nadler et al. [13]. This formula also uses the total body surface, total body mass (in kg) and sex difference for calculating TBV.

$$blood{volume}_{T_{0}}[L]=\{\begin{array}{c} height{\left[m\right]}^{3}\cdot 0.417+weight\left[kg\right]\cdot 0.0450-0.030,\mathrm{for}\;female\;patients\\ height{\left[m\right]}^{3}\cdot 0.414+weight\left[kg\right]\cdot 0.0328-0.030,\mathrm{for}\;male\;patients \end{array}$$

All calculations and statistical analyses were conducted with both the “Nadler” and the “Allen” formulas.

### 2.5. Statistical Analyses

Statistical analyses were conducted using the open-source software R (version 4.3.0, R Core Team 2020—free software for statistical computing and graphics: a language and environment for statistical computing; R Foundation for Statistical Computing, Vienna, Austria). A descriptive overview of all relevant variables was given by providing absolute and relative frequencies for categorical variables (with percentage) and the median together with the 1st and 3rd quartile for continuous variables. In order to analyze the pairwise correlation between the measured variables of interest and their corresponding theoretical variables, both being continuous variables, Pearson’s product moment correlation coefficient was considered. For visual inspection of these associations, scatter plots are presented. Box plots were used for depiction of data at T_0_ and T_1_.

## 3. Results

### 3.1. Patient Characteristics

Between November 2018 and July 2022, a total of 190 adult patients were recruited into the study. For the analysis, 174 patients were eligible. The drop out of 16 patients was due to unplanned extended surgical courses, the implantation of an extracorporeal membrane oxygenation (ECMO) during the operation or incomplete data. The median age of the included patients was 67 (59–74) with a body mass index (BMI) of 26 kg/m^2^ (23.7–29.1) and a cardiac surgical risk (Euroscore II) of 1.44 (0.86–2.52), as shown in Table 1.

### 3.2. Surgical Procedure and Fluid Management

The majority of the patients received valve surgery (56%), which was followed by coronary artery bypass graft surgery (24%) and combined coronary artery bypass graft surgery with valve surgery (20%), as shown in Table 1. The median duration of cardiopulmonary bypass was 143 (111–177) minutes. We assessed the CPB time to compare the different surgical procedures, as the overall fluid management might be related to the duration of surgery. Before the beginning of surgery (T_0_), Hgb, PT and PLT were in the normal ranges (Table 1). At the end of the CPB (T_1_), these parameters were lower (*p* < 0.001 for each parameter between T_0_ and T_1_).

During the surgical procedure with CPB, each patient received comparable amounts of additional volume (500 mL of colloids and 520 mL of crystalloids, Table 2). The applied volumes of packed red blood cells, fresh frozen plasma and platelet concentrates were added with volumes of 280 mL, 250 mL and 300 mL, respectively. The priming volume as well as the cardioplegic solution amounts were assessed together and resulted in an overall median positive fluid balance of 2790 mL (1949–3548) at the end of CPB (FBC).

### 3.3. Calculation of Individual Dilution-Related Volume

**Figure 1:**
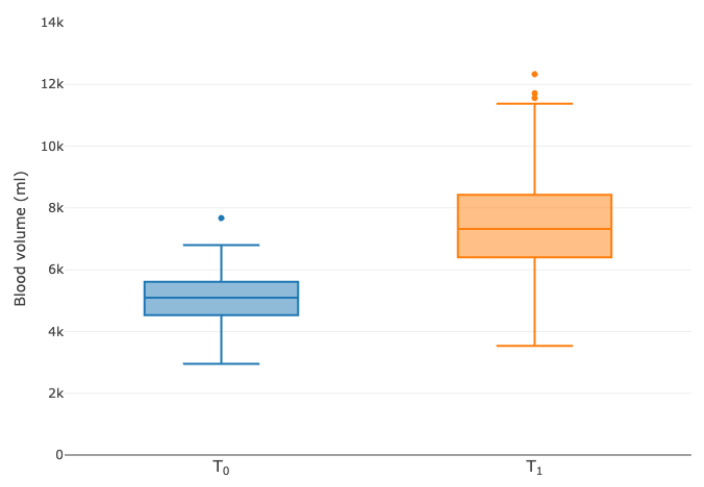
Boxplots of calculated total blood volume for T_0_ and T_1_.

For each patient, the individual dilution-related volume was calculated using the fluid balance added to the TBV calculated according to Nadler et al. (Figure 1) [13]. The dilution-related volume was increased by a median of 48.0% (IQR 32.5–62.1%), resulting in an increased TBV of 148.0%.

**Figure 2:**
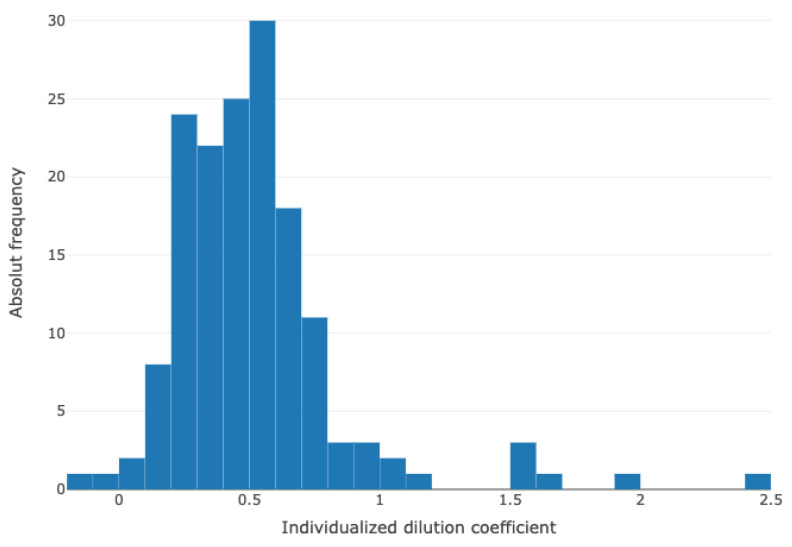
Frequencies of individual dilution coefficients calculated for each patient compared to the total blood volume. Outliers present obese patients with a tendency of a prolonged CPB time.

The dilution coefficient indicates the rate of additional dilution-related volume per TBV before surgery (T_0_, Figure 2).

### 3.4. Correlations between Selected Calculated Diluted and Measured Parameters

**Figure 3:**
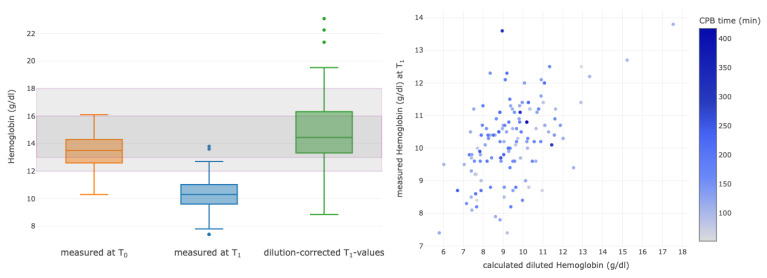
Boxplots of measured values for hemoglobin (g/dL) at baseline (T_0_, orange) and at the end of the CPB (T_1_, blue). The last boxplot displays the values measured at T_1_ after calculated correction of dilution (green). Correlations are shown between the calculated and the measured values of the diluted values of hemoglobin at the end of surgery (T_1_). The colors of the dots represent the CPB time, as outlined in the column on the right side, with the change in color showing different durations of CPB for each patient. CPB, cardiopulmonary bypass.

**Figure 4:**
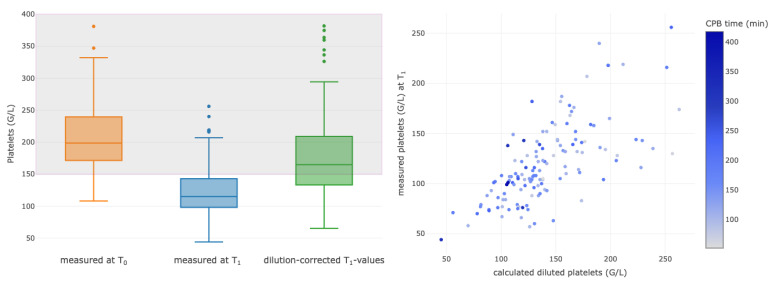
Boxplots of measured values for platelets (G/L) at baseline (T_0_, orange) and at the end of the CPB (T_1_, blue). The last boxplot displays the values measured at T_1_ after calculated correction of dilution (green). Correlations are shown between the calculated and the measured values of the diluted values of platelets at the end of surgery (T_1_). The colors of the dots represent the CPB time, as outlined in the column on the right side, with the change in color showing different durations of CPB for each patient. CPB, cardiopulmonary bypass.

**Figure 5:**
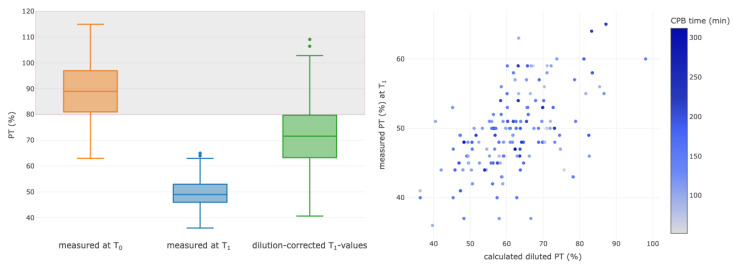
Boxplots of measured values for prothrombin time (%) at baseline (T_0_, orange) and at the end of the CPB (T_1_, blue). The last boxplot displays the values measured at T_1_ after calculated correction of dilution (green). Correlations are shown between the calculated and the measured values of the diluted values of prothrombin time at the end of surgery (T_1_). The colors of the dots represent the CPB time, as outlined in the column on the right side, with the change in color showing different durations of CPB for each patient. CPB, cardiopulmonary bypass.

Hemoglobin levels, PLT and PT were selected for further analyses of hemodilution at the end of CPB (T_1_). To assess the effect of the new dilution coefficient on these laboratory parameters, the parameters at the times T_0_ and T_1_ were compared, and the individual dilution coefficient was back-calculated to estimate a non-diluted value at timepoint T_1_. The laboratory values compared to their baseline values decreased in the median by 24% for Hgb, 41% for PLT and 44% for PT (Figure 3, Figure 4 and Figure 5, left column). For each parameter, correlations between measured and calculated diluted values were assessed (Figure 3, Figure 4 and Figure 5, right column).

The correlation between the calculated dilution value and the measured T_1_ values for Hgb was significant (*p* < 0.001; correlation coefficient (corrCoeff) = 0.53 (95% CI 0.39–0.64), Figure 3, right). Moreover, the majority of the patients (78.7%) did not receive packed red blood cell concentrates, and these patients were included in the final analysis (*n* = 137).

The calculated dilution value and the measured T_1_ platelet count were correlated with a corrCoeff of 0.68 (95% CI 0.58–0.64), *p* < 0.001 (Figure 4, right). The patients who received either platelet concentrates or desmopressin acetat (Octostim™) for possible platelet stimulation were excluded from this analysis. The exact platelet numbers in platelet concentrates may considerably vary, and the effect of desmopressin on the platelet count could not exactly be assessed. Thus, data from 152 patients were eligible for the analysis.

The calculated dilution value and measured PT T_1_ values were correlated with a corrCoeff of 0.55 (95% CI 0.42–0.65), *p* < 0.001 (Figure 5, right). Ten datasets were excluded from the analysis due to transfusion of fresh frozen plasma or usage of a four-factor prothrombin complex concentrate, which could have influenced the absolute PT results.

Validation was performed using the “Allen” formula for the calculation of TBV. Minor differences were observed in all the parameters, but all the statistical tests remained highly significant with *p* < 0.001, and the confidence intervals were also similar: for Hgb, the corrCoeff was = 0.56 (95% CI 0.42–0.67); for PLT, the corrCoeff was 0.69 (95% CI 0.60–0.77) and for PT, the corrCoeff was 0.57 (95% CI 0.44–0.67 (Supplementary Materials)).

## 4. Discussion

The main finding of this prospective observational study from a central European tertiary university center was that the commonly measured laboratory parameters were significantly decreased after weaning from CPB compared to the baseline. The reduction in Hgb levels (24%), PLT (41%) and PT (44%) was correlated with increasing levels of hemodilution. Interestingly, the magnitude of hemodilution was independent of the CPB duration.

The amount of fluid therapy largely varies in patients undergoing cardiac surgery with the installation of CPB. Most studies report hematocrit values as a surrogate parameter for assessing patients’ hemodilution status, but they do not report details on additional volume substitution or urine output [5,14,15]. This approach seems to be simplified and should not be considered as reliable. In a further study, a volume-based calculated approach for the assessment of hemodilution was compared against a simple estimation of volume load from an experienced anesthesiologist [16]. Interestingly, both methods could not reliably predict the level of periinterventional hemodilution. As a consequence, the level of hemodilution is not a routinely assessed parameter in cardiac surgery patients, despite its possible implications for concentration-based laboratory parameters such as Hgb values, PT or PLT. This may lead to the unnecessary substitution of packed red blood cell concentrates, platelet concentrates and fresh frozen plasma or coagulation factor concentrates.

Nowadays, the effect of dilution on laboratory parameters cannot be measured in standard institutional care, and the level of hemodilution remains the subject of debate. Nevertheless, the continuous intraoperative monitoring of laboratory parameters and the potential substitution of Hgb or PLT is important for maintaining oxygen supply and hemostasis throughout surgery and in the early postoperative course. In clinical situations, where large amounts of intravenous fluids are needed, concentration-based laboratory parameters can only provide values that are not corrected for dilution. Moreover, these parameters are usually used to guide therapeutical decisions, for example, the substitution of packed red blood cells or PLT concentrates, to meet the accepted and predefined transfusion thresholds. In an earlier randomized controlled trial of patients undergoing cardiac surgery, the use of a restrictive perioperative transfusion strategy compared with a more liberal approach did not result in an outcome difference in terms of 30-day all-cause mortality or severe morbidity [17]. One of the reasons for this could also be the effect of dilution.

In our study, we present a new approach to assess the level of hemodilution based on Hgb, PLT and PT values that can be used to calculate a patient-specific dilution coefficient. This approach is newly developed and can provide a reliable quantitative assessment of patients’ dilution status in cardiac surgery. The “Nadler” formula for the calculation of TBV was chosen because this formula had previously been validated using radioactive-labeled I—131 albumin in 155 healthy adults, showing a better conformity in estimating circulating TBV than the older “Allen” formula, which is based on body surface and total body mass (in kilograms) [13]. We did not apply other approaches using body height or body weight without an additional constant, as they were not validated using radioactive tracers.

The rapid postoperative normalization of the coagulation system is essential to avoid bleeding complications, which are observed in up to 22% of postcardiotomy patients [18]. A low postoperative Hgb leads to increased morbidity and mortality and a prolonged ICU and hospital stay [19]. Standard laboratory parameters like Hgb, PLT and PT are routinely used to estimate the need for transfusion or coagulation therapy. As these parameters are easily available in all routine diagnostic laboratories, we integrated their values in our calculation. We showed that the integration of a correction factor for dilution for each of the concentration-based laboratory variables yielded reliable results. It could be speculated that the calculated values may better reflect the actual concentration of Hb, Hct and PT, which may be used to estimate the need for the transfusion of coagulation therapy.

### 4.1. Future Perspectives

To the best of our knowledge, no technical device for a continuous or point-of-care modality for the measurement of total plasma volume has been available so far. For experimental use, dilution agents like 99mTc-labelled serum albumin or Evans blue are used to determine the plasma volume, but this approach is not feasible in clinical daily routine or for critically ill patients [20,21]. Most of the available measurement devices serve to monitor the fluid status and fluid responsiveness of the patient, but they cannot provide information on the actual intravasal or plasma volume [22,23]. Here, the presented dilution formula can help in the estimation of the intravasal volume without the need for invasive diagnostic modalities or devices, and it can give important information about the influence of dilution on concentration-based laboratory values in a short time. To date, no strong evidence on transfusion thresholds or methods for exact blood loss monitoring, hemodilution and decision-making regarding blood product substitutions are available, especially in case of interventions with significant fluid changeover.

### 4.2. Strengths and Limitations

An important strength of our study is, besides its prospective nature, the large number of patients with the assessment of both laboratory parameters and fluid balances and the clear definition of the included patients. Our prospective cohort only included patients undergoing elective cardiac surgery, as shown using the low average Euroscore II [12], and a sex distribution comparable to other cardiac surgical patient collectives [24]. Patients with an increased risk of a complex surgical course (e.g., valvular repair or active endocarditis) were not included. Furthermore, patients with acute myocardial infarction, with hyperinflammation accompanied by vasoplegia after CPB [25] and the need for additional fluid therapy for a longer period were excluded from our study.

However, our work also has certain limitations. We were not able to continuously measure the amount of urine output; we were only able to measure it at the end of surgery. Therefore, a time-dependent approach was used to calculate the amount of excreted urine. Clinical experience shows that diuresis changes during a surgical procedure (lower amounts at the beginning of surgery and increasing amounts after weaning from CPB and/or therapy with diuretics). This fact could have led to an overestimation of the total diuresis during CPB. However, in this case, the calculated dilution would have been even more pronounced than what we expected. Moreover, the loss of fluid through intraoperative ventilation, perspiration and humidification could not be assessed and was therefore not included in our calculation [26,27,28,29]. Finally, it was not possible to assess blood loss at the end of CPB until 15 min after the end of protamine infusion. However, the blood loss during this time was considered to be low, as the time interval between the end of CPB and blood withdrawal for the assessment of blood gases was short (20 min).

## 5. Conclusions

Based on the individual data from fluid balances and diuresis, hemodilution affects the levels of hemoglobin, the platelet count and the prothrombin time by a median of 43%. Such calculations could be useful to better estimate the individual amount of dilution in patients undergoing cardiac surgery with CPB. Moreover, using our formula, we could finally better understand when a potential transfusion trigger was reached. Further research on the impact of dilution and related decision-making for blood product substitution, including its impact on morbidity and mortality, is warranted.

## Figures and Tables

**Table 1:** Demographic and clinical characteristics of analyzed population.

Characteristics	Patient Data (*n* = 174)
Age (years)	67 (59–74)
Female sex (%)	52 (29.9)
Height (cm)	174.5 (168–180)
Weight (kg)	80 (70–90)
Body mass index (kg/m^2^)	26.1 (23.7–29.1)
Euroscore II	1.44 (0.86–2.52)
Valve surgery	97 (56%)
Coronary artery bypass graft surgery	41 (24%)
Combined CABG with valve surgery	36 (20%)
CPB duration (minutes)	143 (111–177)
**Laboratory measurements**	
Hemoglobin T_0_ (g/dL)	12.9 (11.6–14.2)
Prothrombin time T_0_ (%)	89 (81–97)
Platelet count T_0_ (G/L)	198 (168–237)
Hemoglobin T_1_ (g/dL)	10.1 (9.4–10.8)
Prothrombin time T_1_ (%)	49 (45–52)
Platelet count T_1_ (G/L)	113 (94–143)

CABG, coronary artery bypass graft surgery; CPB, cardiopulmonary bypass.

**Table 2:** Fluid balance.

Fluid Balance	Patient Data (*n* = 174)
**Infusions**	
Colloids (mL)	500 (500–500)
Crystalloids (mL)	520 (500–900)
CPB positive fluid balance change (mL)	2790 (1949–3548)
Packed red blood cells units (280 mL each)	
One concentrate	16 (9%)
Two concentrates	13 (7%)
≥Three concentrates	8 (5%)
Platelet concentrate units (300 mL each)	13 (8%)
Desmopressin	9 (5%)
**Diuresis** (estimated in mL)	1016 (712–1541)

CPB, cardiopulmonary bypass.

## Data Availability

The datasets used and analyzed during the current study can be made available from the corresponding author on reasonable request.

## Supplementary Materials

The following supporting information can be downloaded at: https://www.mdpi.com/article/10.3390/diagnostics13152596/s1, Figure S1: Boxplots of calculated total blood volume for T0 and T1. Figure S2: Frequencies of individual dilution coefficients calculated for each patient compared to the total blood volume. Figure S3: Boxplots of measured values for hemoglobin (g/dL) at baseline (T0, orange) and at the end of the CPB (T1, blue). The last boxplot displays the values measured at T1 after calculated correction of dilution (green). Correlations are shown between the calculated and the measured values of the diluted values of hemoglobin at the end of surgery (T1). The colors of the dots represent the CPB time as outlined in the column on the right side, with the change of color showing different durations of CPB for each patient. CPB, cardiopulmonary bypass. Figure S4: Boxplots of measured values for platelets (G/L) at baseline (T0, orange) and at the end of the CPB (T1, blue). The last boxplot displays the values measured at T1 after calculated correction of dilution (green). Correlations are shown between the calculated and the measured values of the diluted values of platelets at the end of surgery (T1). The colors of the dots represent the CPB time as outlined in the column on the right side, with the change of color showing different durations of CPB for each patient. CPB, cardiopulmonary bypass. Figure S5. Boxplots of measured values for prothrombin time (%) at baseline (T0, orange) and at the end of the CPB (T1, blue). The last boxplot displays the values measured at T1 after calculated correction of dilution (green). Correlations are shown between the calculated and the measured values of the diluted values of prothrombin time at the end of surgery (T1). The colors of the dots represent the CPB time as outlined in the column on the right side, with the change of color showing different durations of CPB for each patient. CPB, cardiopulmonary bypass.
