# Supplementary material for: Loss or Dilution—A New Diagnostic Method to Assess the Impact of Dilution on Standard Laboratory Parameters
Source: Diagnostics (Basel). 2023 Aug 4;13(15):2596. doi: 10.3390/diagnostics13152596 (PMC10417005; doi:10.3390/diagnostics13152596)
Supplement: Supplementary file 1 [file diagnostics-13-02596-s001.zip › diagnostics-2502646-supplementary.pdf]

Calculations with Allen Formula for total blood volume (TBV, in ml):

$$bloodvolume_{T_0}[l] = \begin{cases} height[m]^3 \cdot 0.417 + weight[kg] \cdot 0.0450 - 0.030, \text{for } female\ patients \\ height[m]^3 \cdot 0.414 + weight[kg] \cdot 0.0328 - 0.030, \text{for } male\ patients \end{cases}$$

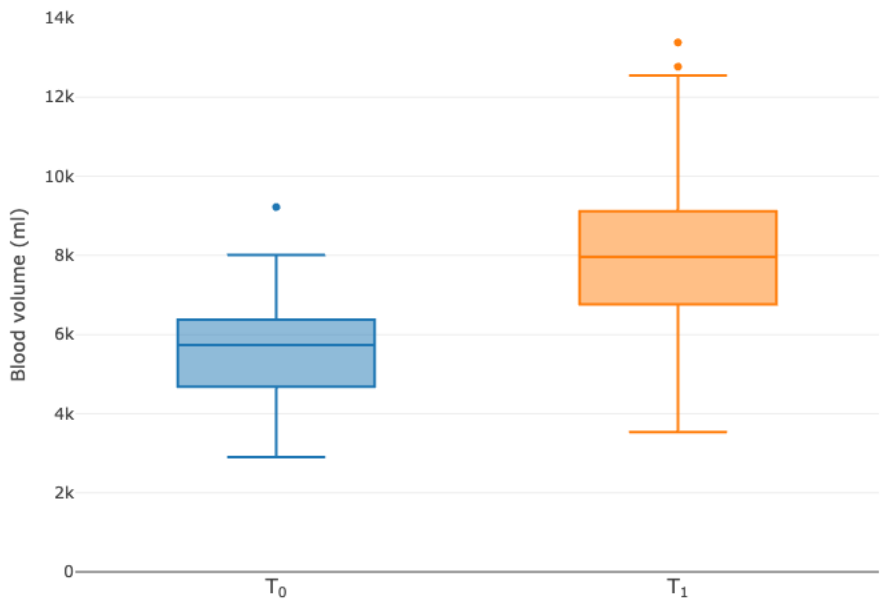


**Figure S1.** Boxplots of calculated total blood volume for $T_0$ and $T_1$.

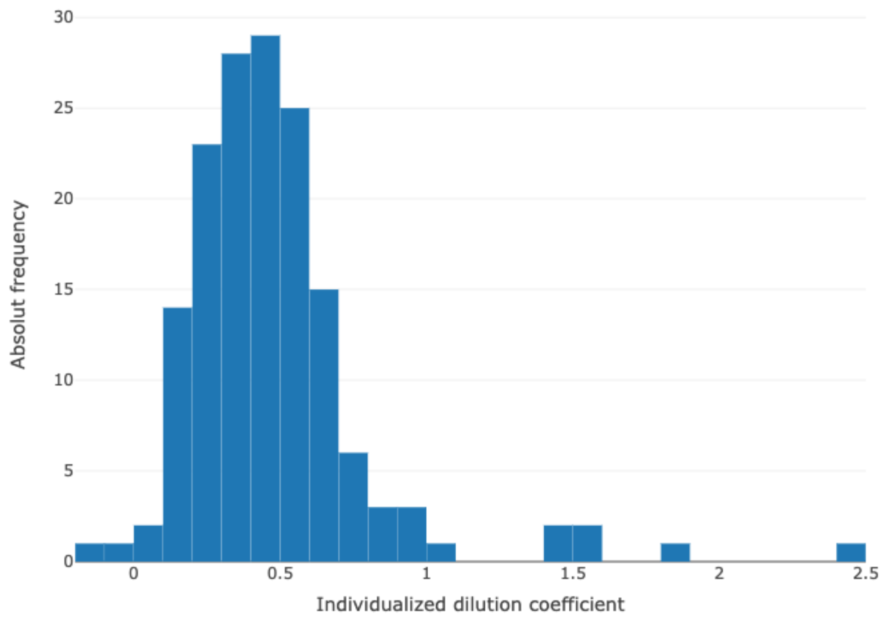


**Figure S2:** Frequencies of individual dilution coefficients calculated for each patient compared to the total blood volume.

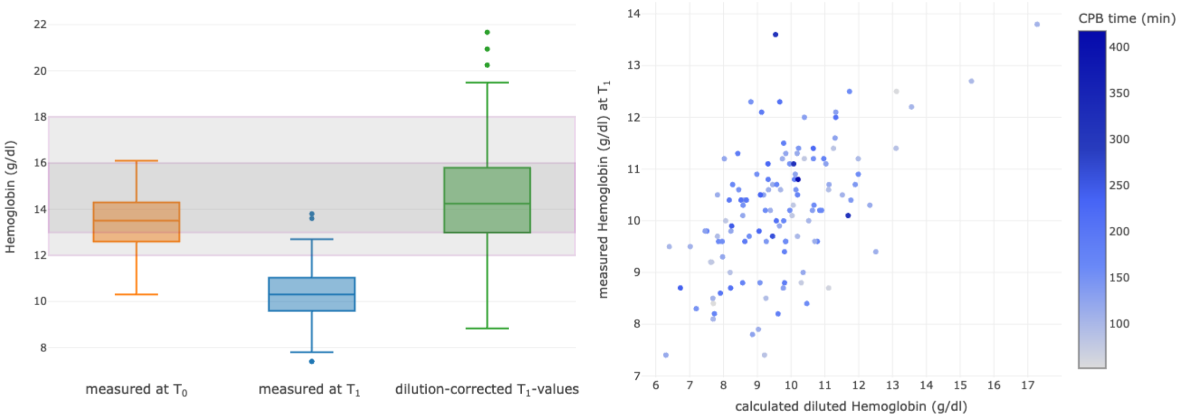


**Figure S3.** Boxplots of measured values for hemoglobin (g/dl) at baseline ($T_0$, orange) and at the end of the CPB ($T_1$, blue). The last boxplot displays the values measured at $T_1$ after calculated correction of dilution (green). Correlations are shown between the calculated and the measured values of the diluted values of hemoglobin at the end of surgery ($T_1$). The colors of the dots represent the CPB time as outlined in the column on the right side, with the change of color showing different durations of CPB for each patient. CPB, cardiopulmonary bypass

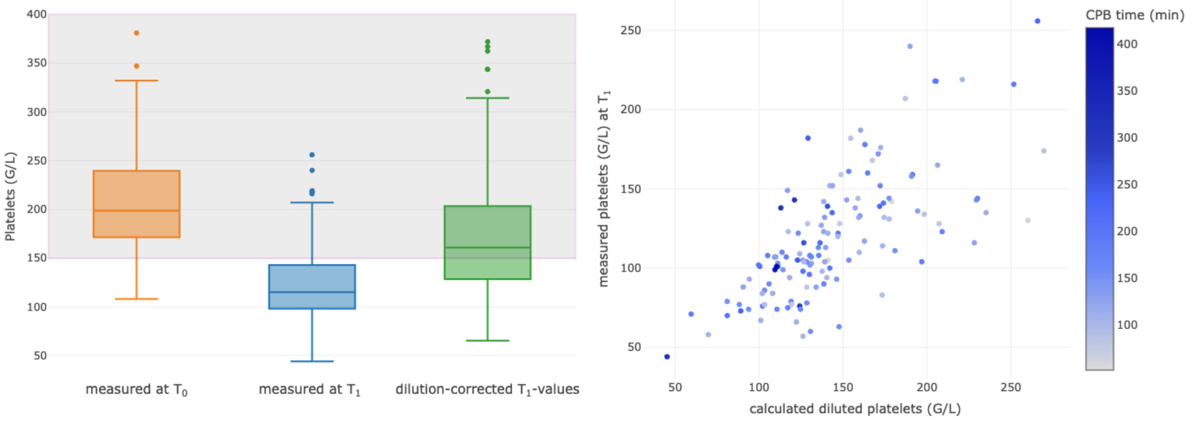


**Figure S4.** Boxplots of measured values for platelets (G/L) at baseline ($T_0$, orange) and at the end of the CPB ($T_1$, blue). The last boxplot displays the values measured at $T_1$ after calculated correction of dilution (green). Correlations are shown between the calculated and the measured values of the diluted values of platelets at the end of surgery ($T_1$). The colors of the dots represent the CPB time as outlined in the column on the right side, with the change of color showing different durations of CPB for each patient. CPB, cardiopulmonary bypass

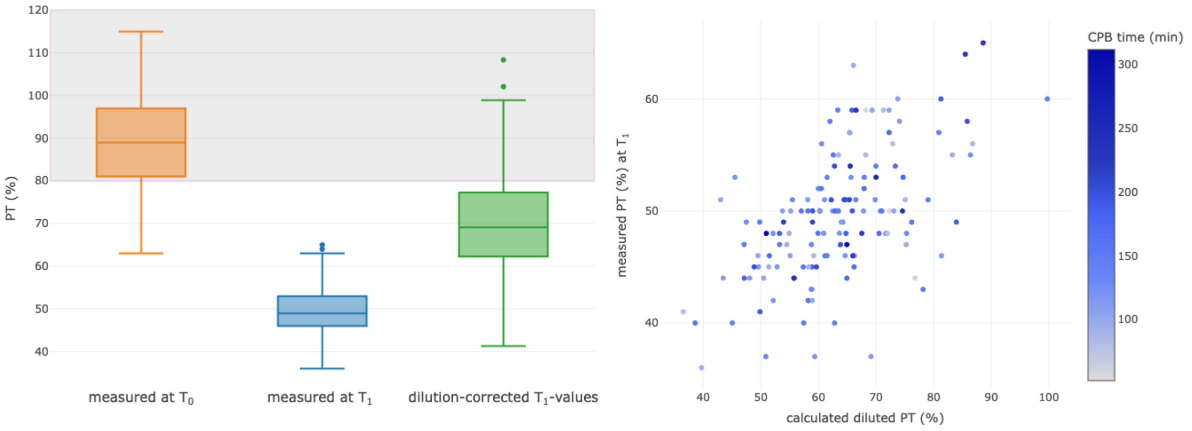


**Figure S5.** Boxplots of measured values for prothrombin time (%) at baseline ($T_0$, orange) and at the end of the CPB ($T_1$, blue). The last boxplot displays the values measured at $T_1$ after calculated correction of dilution (green). Correlations are shown between the calculated and the measured values of the diluted values of prothrombin time at the end of surgery ($T_1$). The colors of the dots represent the CPB time as outlined in the column on the right side, with the change of color showing different durations of CPB for each patient. CPB, cardiopulmonary bypass
